# Supplementary figures and images for: Fibroblast mechanoperception instructs pulmonary developmental and pattern specification gene expression programs
Source: PLoS Genet. 2025 Nov 10;21(11):e1011924. doi: 10.1371/journal.pgen.1011924 (PMC12633902; doi:10.1371/journal.pgen.1011924)

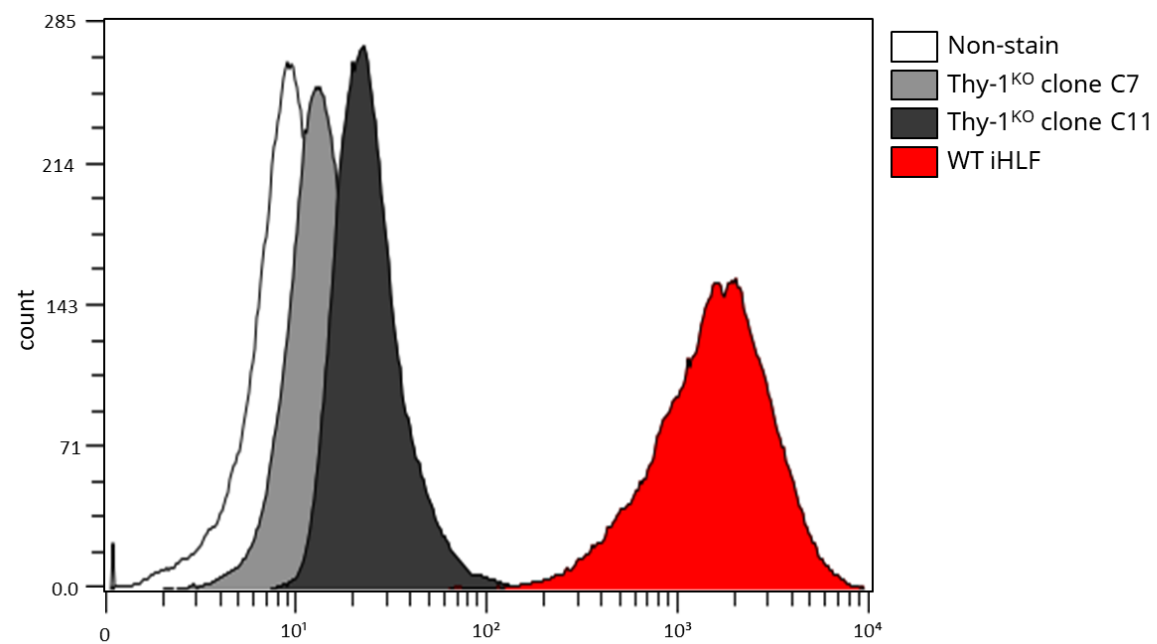

Supplement: S1 Fig — (PDF) [file pgen.1011924.s001.pdf]

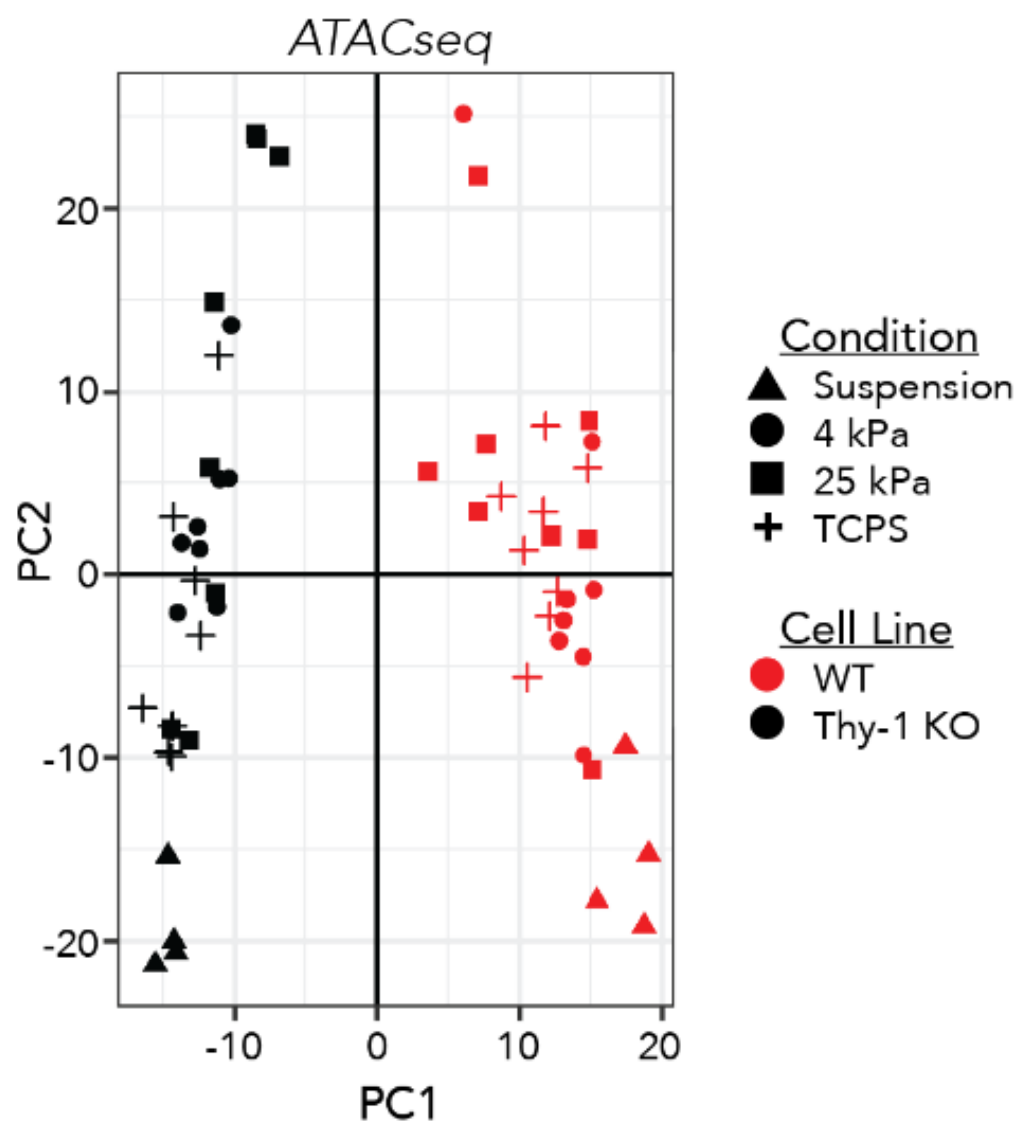

Supplement: S2 Fig — X and Y-axes represent the primary and secondary principal components (PC1, PC2), respectively. PC1 = 48.20% of variance, PC2 = 36.68% of variance. (PDF) [file pgen.1011924.s002.pdf]

**A**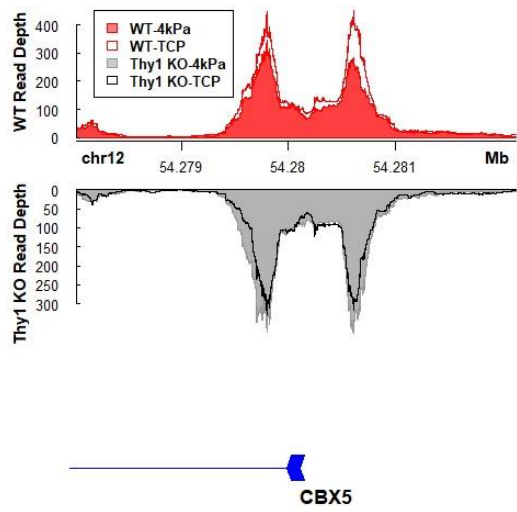**B**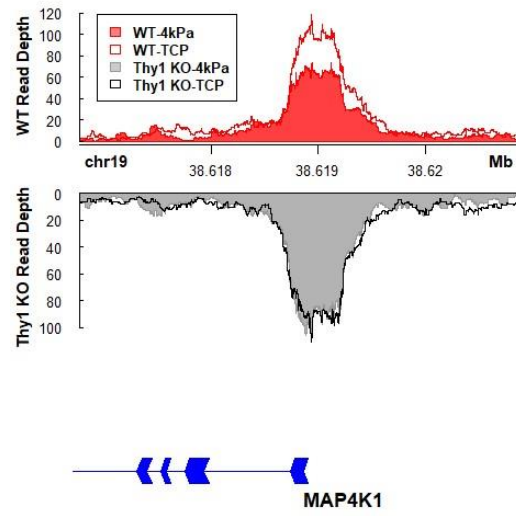**C**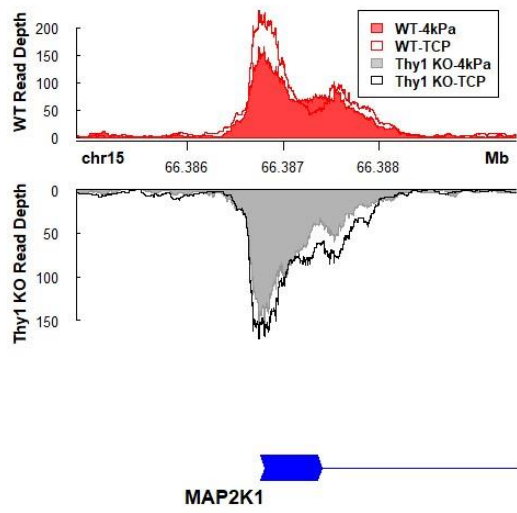

Supplement: S3 Fig — Opaque traces represent 4kPa stiffness conditions; transparent traces represent 1GPa/TCP conditions. (PDF) [file pgen.1011924.s003.pdf]

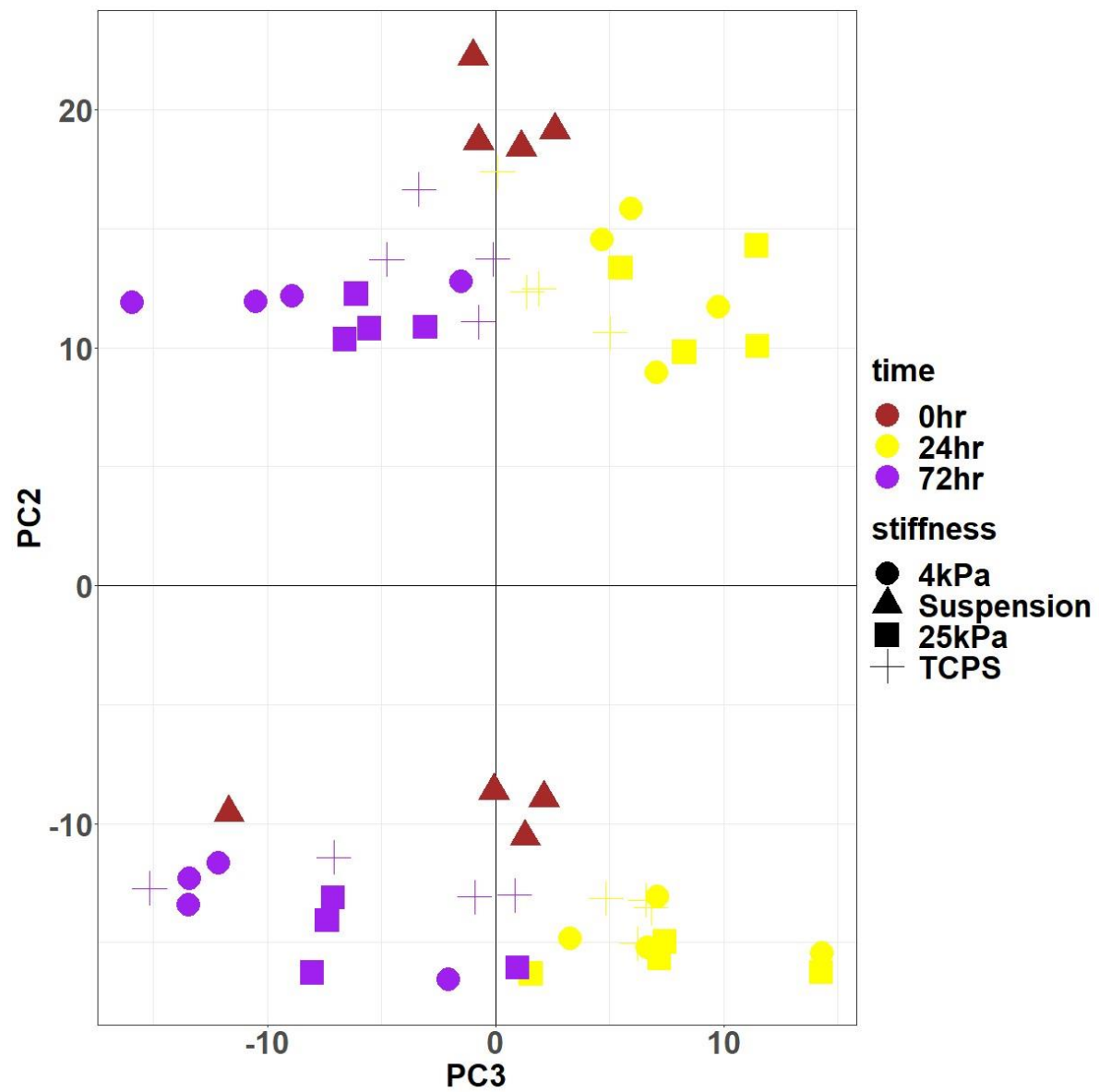

Supplement: S4 Fig — Top cluster represents WT samples while bottom cluster represents Thy-1KO samples. PC2 = 36.68% of variance, PC3 = 4.66% of variance. (PDF) [file pgen.1011924.s004.pdf]

A

*WT vs Thy-1 KO (72hr)*  
*Differentially Expressed Genes*

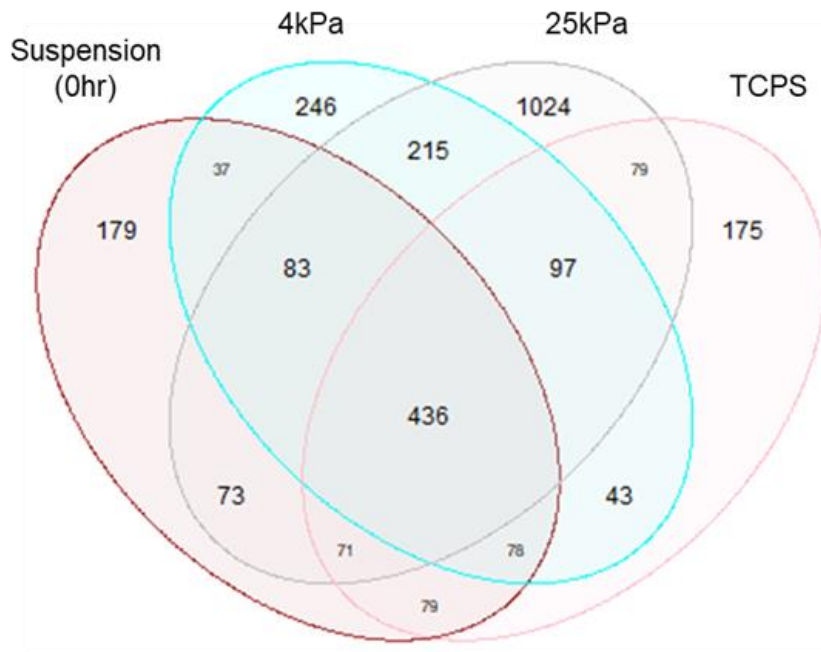

B

*WT vs Thy-1 KO*  
*Differentially Expressed Genes*

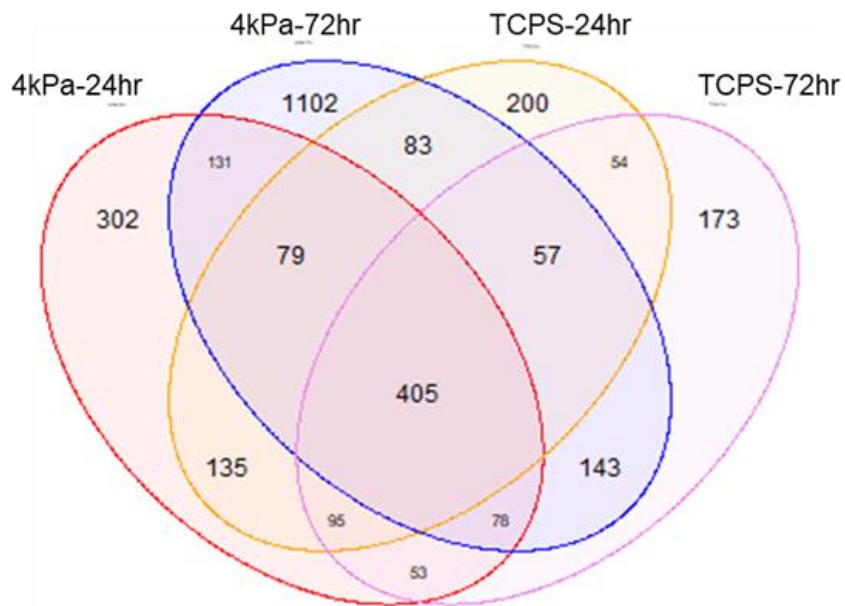

Supplement: S5 Fig — In all cases a conserved set of genes displayed significant changes in expression after Thy-1 loss (Wald test with BH correction; p < 0.05). (PDF) [file pgen.1011924.s005.pdf]

### Suspension (0hr)

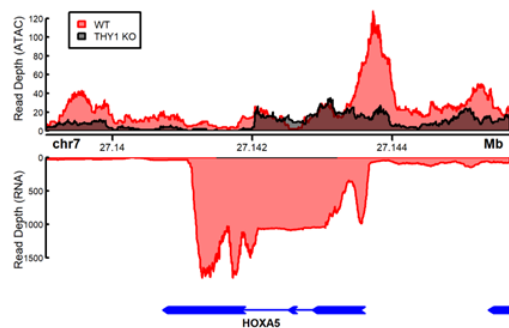

### 4kPa – 72hr

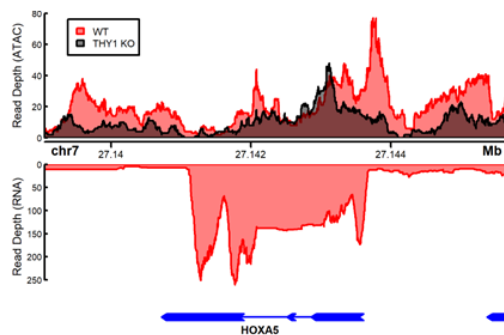

### 25kPa – 24hr

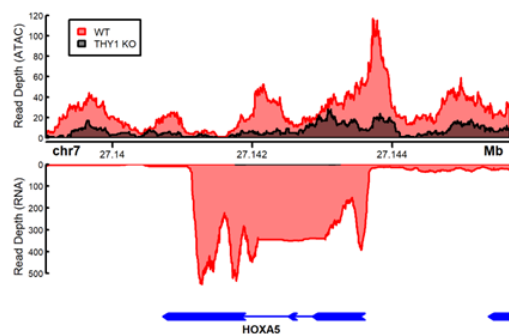

### 25kPa – 72hr

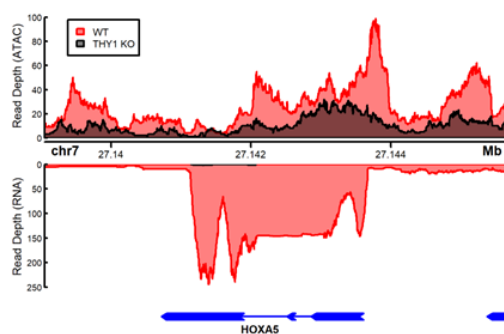

### TCPS – 24hr

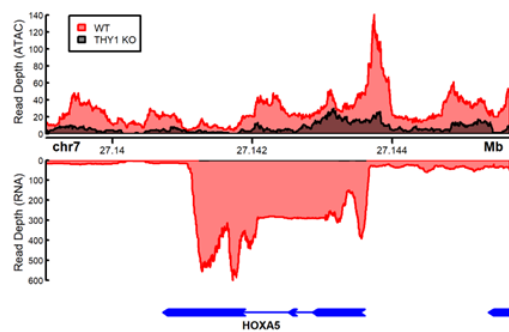

### TCPS – 72hr

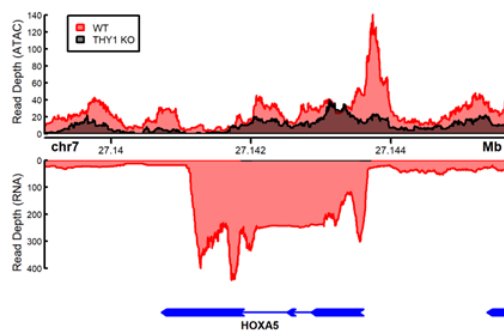

Supplement: S6 Fig — Top panel represents ATAC-seq data; bottom panel represents RNA-seq data. (PDF) [file pgen.1011924.s006.pdf]

## HOXB5: WT vs. Thy-1 KO

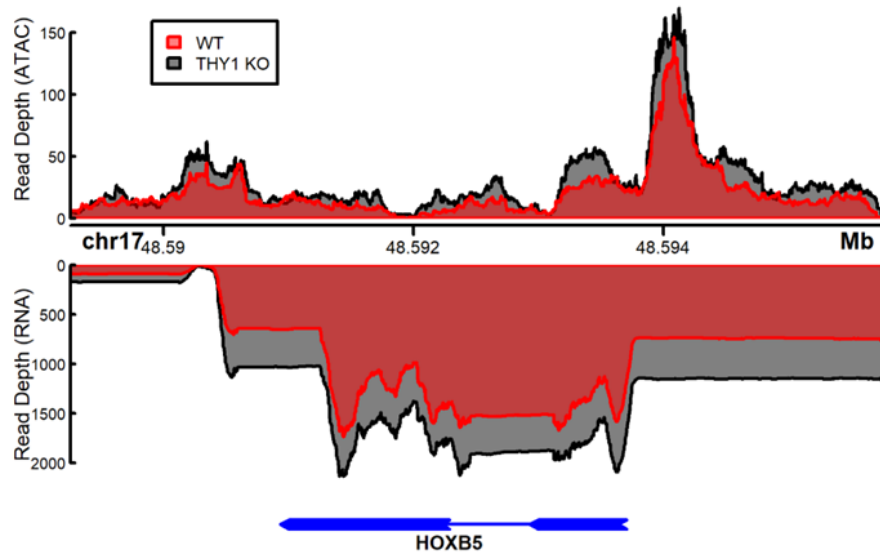

## HOXC5: WT vs. Thy-1 KO

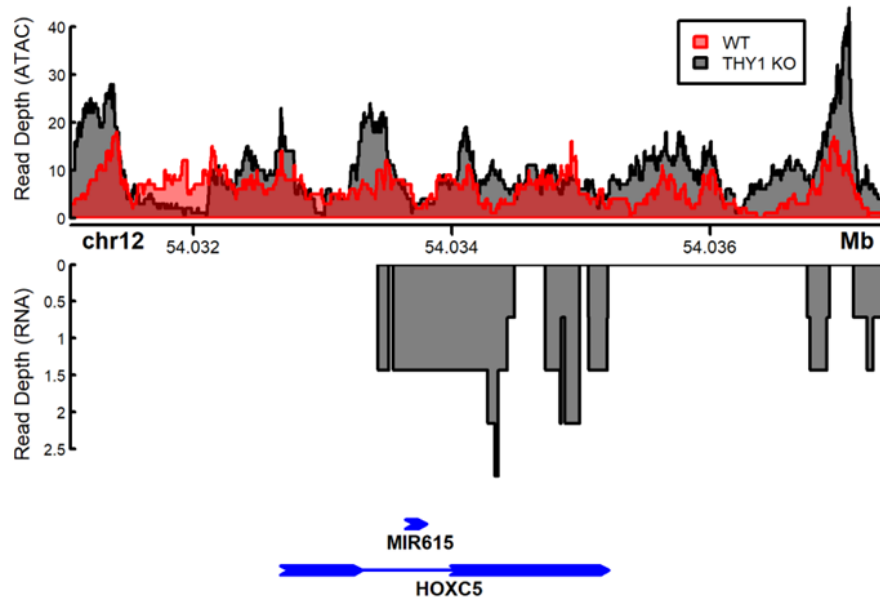

Supplement: S7 Fig — Top panel represents ATAC-seq data; bottom panel represents RNA-seq data. No RNA-seq data is shown for WT sample at the HOXC5 locus due to an absence of detectable RNA signal. (PDF) [file pgen.1011924.s007.pdf]

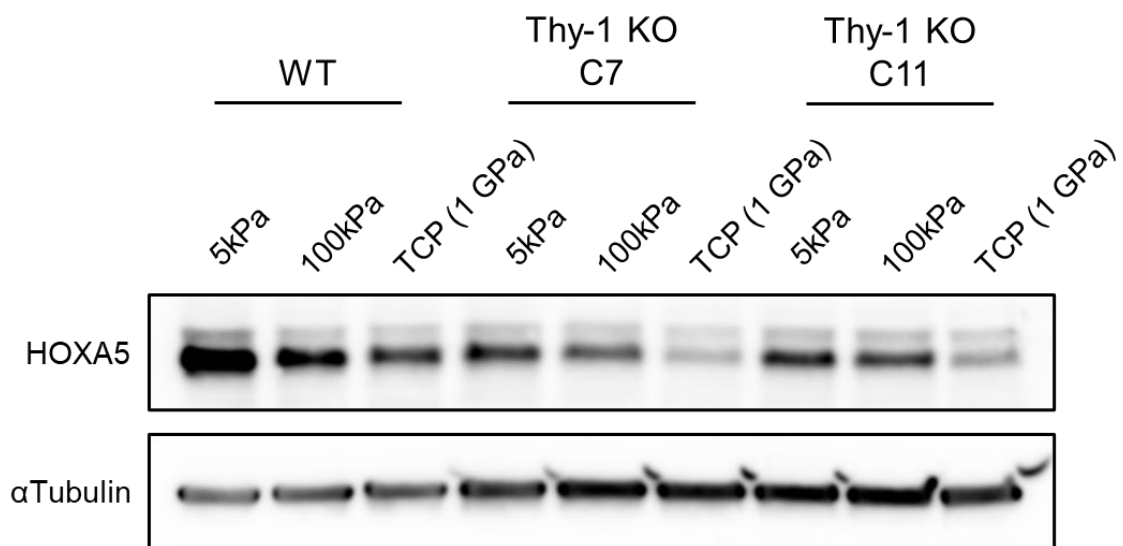

Supplement: S8 Fig — (PDF) [file pgen.1011924.s008.pdf]

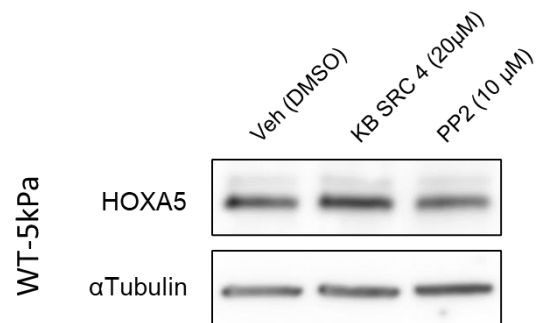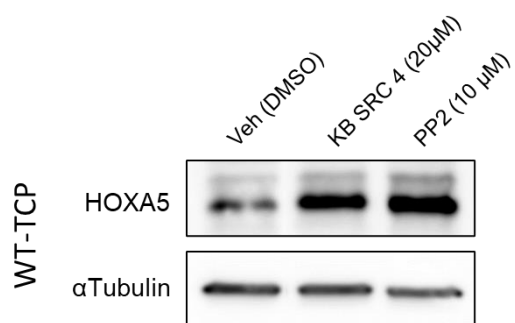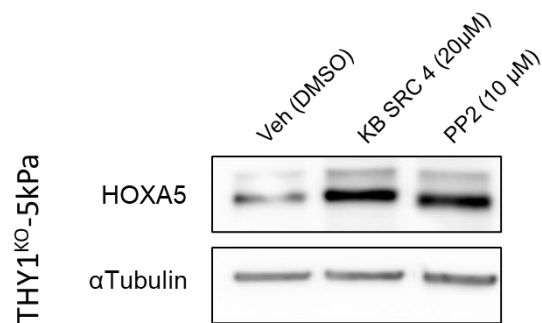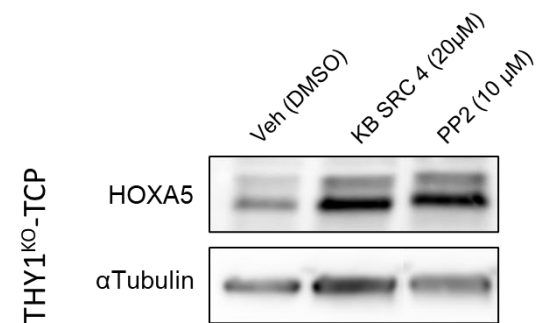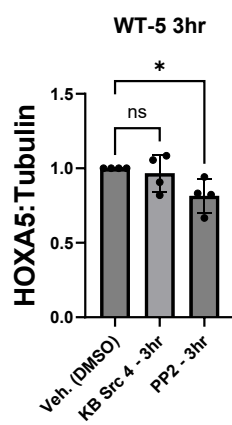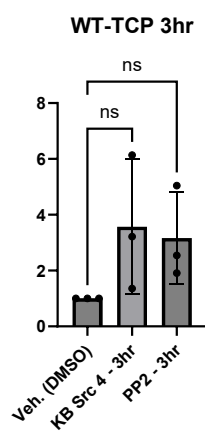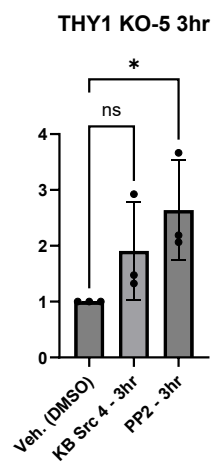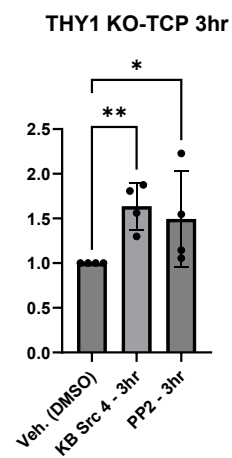

Supplement: S9 Fig — Samples were allowed to adhere overnight then treated with 10μM pan-SRC family kinase (pan-SFK) inhibitor PP2 or 20μM SRC inhibitor KB SRC 4 for 3h. Plots represent ratio of signal to vehicle condition after normalization of HOXA5 signal to loading control. Mean ± S.D. plotted; N = 3 (Thy-1KO-5kPa and WT-1GPa/TCP) or N = 4 (WT-5kPa and Thy-1KO-1GPa) independent experiments; WT-5kPa, Thy-1KO-5kPa and Thy-1KO-1GPa = non-parametric Kruskal-Wallis test with post-hoc uncorrected Dunn’s test and WT-1GPa = 1-way ANOVA with post-hoc uncorrected Fisher’s LSD test. Tests chosen based on data distribution and variance. For all statistical tests: ns = p > 0.05; p < 0.05 (*); p < 0.01 (**); p < .001 (***); p < .0001 (****). (PDF) [file pgen.1011924.s009.pdf]

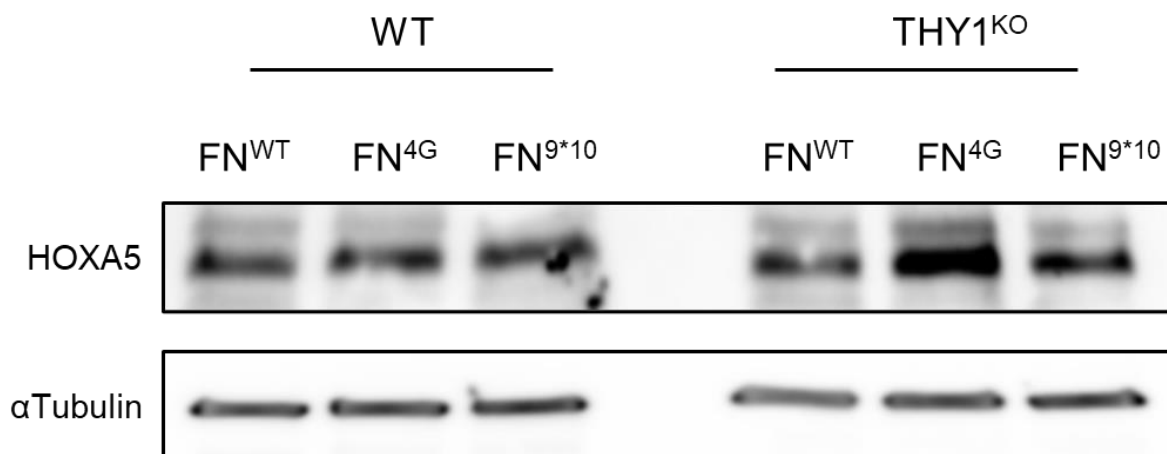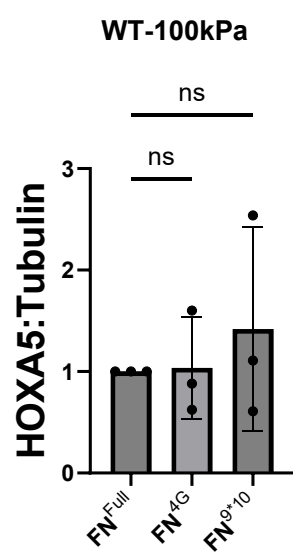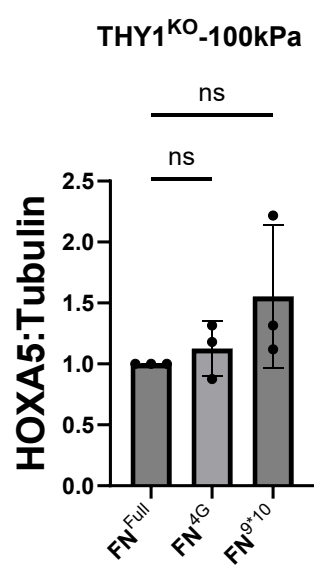

Supplement: S10 Fig — No significant changes in HOXA5 expression were identified. Plots represent ratio of signal to full-length condition after normalization of HOXA5 signal to loading control. Mean ± S.D. plotted; N = 3 independent experiments; WT-100kPa = 1-way ANOVA with post-hoc uncorrected Fisher’s LSD test, Thy-1KO-100kPa = Brown-Forsythe and Welch ANOVA with post-hoc unpaired t-tests with Welch’s correction. For all statistical tests: ns = p > 0.05; p < 0.05 (*); p < 0.01 (**); p < .001 (***); p < .0001 (****). (PDF) [file pgen.1011924.s010.pdf]
