# Supplementary material for: Fibroblast mechanoperception instructs pulmonary developmental and pattern specification gene expression programs
Source: PLoS Genet. 2025 Nov 10;21(11):e1011924. doi: 10.1371/journal.pgen.1011924 (PMC12633902; doi:10.1371/journal.pgen.1011924)
Supplement: S11 Fig — E < 1e-3 was applied to determine a significant change in global binding site accessibility. (PDF) [file pgen.1011924.s011.pdf]

HOXA5

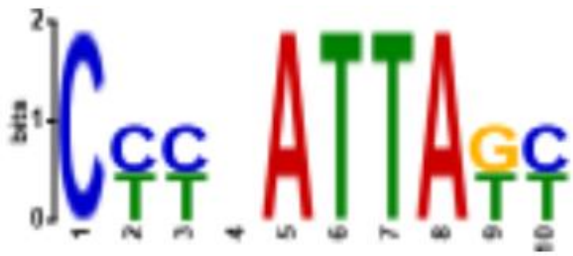

(Consensus Motif –CYYNATTAKY)

Test Condition	Background Condition	*E*-value
WT-4kPa-24hr	Thy-1 KO-4kPa-24hr	**< 2.09e-11**
WT-TCP-24hr	Thy-1 KO-TCP-24hr	**< 1.59e-2**
